# Descriptive characteristics of prostate cancer in patients with a history of primary male breast cancer – a SEER analysis

**DOI:** 10.1186/s12885-017-3640-7

**Published:** 2017-09-25

**Authors:** Nikita Abhyankar, Kent F. Hoskins, Michael R. Abern, Gregory S. Calip

**Affiliations:** 1 Department of Urology, University of Illinois at Chicago, 820 South Wood Street MC 955, Chicago, IL 60612 USA; 2 Division of Hematology and Oncology, University of Illinois at Chicago, 840 South Wood Street MC 713, Chicago, IL 60612 USA; 3 Department of Pharmacy Systems, Outcomes and Policy, University of Illinois at Chicago, 833 South Wood Street MC 871, Chicago, IL 60612 USA; 4 Center for Pharmacoepidemiology and Pharmacoeconomic Research, University of Illinois at Chicago, 833 South Wood Street MC 871, Chicago, IL 60612 USA

**Keywords:** Male breast cancer, Prostate cancer, Seer, Risk factors

## Abstract

**Background:**

Current evidence on risk of prostate cancer following a diagnosis of male breast cancer is limited and guidance for screening in this potentially higher-risk population remainsunclear. Our objective was to quantify prostate cancer risk in men diagnosed with breast cancer.

**Methods:**

We identified men diagnosed with first primary breast cancer between 1988 and 2012 using the Surveillance, Epidemiology and End Results Program registry databases. Men were followed for occurrence of a second primary prostate cancer and secondary outcomes of cancer-specific and overall survival. Stratified analyses were performed by age, breast cancer stage, race, and breast cancer hormone receptor status. Excess risk per 10,000 person-years and standardized incidence ratios (SIR) with 95% confidence intervals (95% CI) were calculated. We used multivaraible Cox proportional hazard models to estimate hazard ratios (HR) and 95% CI for characteristics associated with secondary prostate cancer and survival.

**Results:**

From a cohort of 5753 men with breast cancer with median follow up of 4.3 years, we identified 250 cases of second primary prostate cancer. Overall, the incidence of second primary prostate cancer was modestly greater than expected (SIR = 1.12, 95% CI 0.93–1.33), although not statistically significant. Stratified analyses demonstrated associations for men ages 65–74 at the time of breast cancer diagnosis (SIR = 1.34, 95%CI 1.01–1.73), hormone receptor-positive breast cancer (SIR = 1.23, 95%CI 1.11–1.39) or AJCC stage I breast cancer (SIR = 1.36, 95%CI 1.04–1.75) and second primary prostate cancer diagnosis.

**Conclusions:**

The incidence of prostate cancer in men with history of breast cancer is similar to the general population. Men with favorable characteristics of their breast cancer were more likely to develop prostate cancer, possibly due to a lower competing risk of breast cancer mortality.

## Background

Male breast cancer is a rare entity, representing only 1% of all breast cancers and 0.003% of male cancers [1]. Prostate cancer on the other hand is the most common male cancer in the U.S. and second leading cause of cancer death [2]. Known risk factors for these hormonally dependent cancers are not overlapping, with the exception of rare individuals harboring susceptibility alleles in the *BRCA2* (and less commonly *BRCA1*) gene. Risk factors for male breast cancer include a first degree relative with breast cancer, obesity, gynecomastia, Klinefelter syndrome, orchitis/epididymitis and low physical activity [3, 4]. Prostate cancer risk factors include family history of prostate cancer, African American race, and age [5].

Male breast cancer and prostate cancer have both been associated with germline mutations in the *BRCA1/2* tumor suppressor genes [6, 7]. *BRCA2* mutations have been shown in one study, which did not select for family history, to have a prevalence of 14% in a male breast cancer population [8]. In men under 65 years of age, carriers of mutated *BRCA1* have a 1.8 fold increased risk of prostate cancer [9] and *BRCA2* carriers an 8.6 fold increased risk [10]. Given the cost of DNA testing and the low prevalence of *BRCA* mutations in the general population (with the exception of individuals with Ashkenazi Jewish ancestry) [11], routine screening of asymptomatic men in the general population for a BRCA mutation as a population-based approach to identify prostate cancer risk cannot be recommended at this time. Prostate specific antigen (PSA) use for screening of prostate cancer in the general population is a contentious issue and it is important to be able to identify potential high-risk populations with adequate life expectancy in whom screening would be indicated. The IMPACT study showed that prostate cancer screening in the *BRCA* population, given their increased risk and mortality, is indicated. PSA > 3.0 was found to have a 48% positive predictive value for prostate cancer in the *BRCA2* population compared to 24% in controls who were not carriers of *BRCA1/2* mutations [12]. This trial also showed a significantly higher rate of detecting intermediate to high-risk prostate cancer when BRCA2 men were screened.

Due to the rarity of male breast cancer, limited data is available regarding its association with other malignancies. Primary male breast cancer has been associated with cancers at sites other than prostate, with a 34% excess risk of any second cancer [13]. Conversely, primary prostate cancer has been associated with a decrease in the overall incidence of second cancer [14]. It has however been associated with an increase risk of specific other cancers namely; bladder, kidney, soft tissue and endocrine cancers [14]. Available literature on the association between male breast and prostate cancer is conflicting, hence further research is necessary. A 2002 Surveillance, Epidemiology and End Results (SEER) data review found no significant increase in prostate cancer (SIR = 1.09, 95% CI 0.85–1.37) for patients with a primary male breast cancer from 1973 to 1996 [15]. On the other hand, an international multicenter study of second primary cancers using cancer registries over 25 years was reported in 2005 [13], which did show a significantly increased incidence of prostate cancer in patients with a history of male breast cancer (SIR = 1.61, 95% CI 1.34–1.93). The SIR was shown to be the highest within 12 months of the breast cancer diagnosis (SIR = 1.94, 95%CI 1.17–3.02). This data raises the possibility that shared risk factors may exist in addition to rare, highly penetrant susceptibility alleles like *BRCA2*. Additional research is needed to clarify a possible etiologic link between these two hormonally-dependent cancers. While routine PSA screening for men at general population risk is controversial, identification of groups of men at higher risk for prostate cancer is of interest. Therefore we sought to further identify the risk of prostate cancer in men who had breast cancer and the characteristics of these patients in order to guide screening in these patients.

## Methods

### Patients and methods

#### Study population and data sources

We performed a retrospective cohort study of men diagnosed with first primary stages I-III breast cancer between 1988 and 2012 from the eighteen population-based cancer registries in the Surveillance, Epidemiology and End Results (SEER) Program, including San Francisco-Oakland, Connecticut, Detroit, Hawaii, Iowa, New Mexico, Seattle-Puget Sound, Utah, Georgia (Metropolitan Atlanta, Rural and Greater Georgia), San Jose-Monterey, Los Angeles, Alaska, Greater California (excluding SF/SJM/LA), Kentucky, Louisiana and New Jersey. Our study included men ages 20 years and older with a confirmed first primary breast cancer with malignant behavior, excluding those with breast cancer identified by death certificate or autopsy only. Similarly, we identified second primary cancers with documentation of histologically confirmed malignant disease. SEER*Stat statistical software version 8.2.1 [16] was used to obtain demographic and clinical information for first primary breast cancer patients: (i) demographic characteristics; (ii) tumor characteristics including American Joint Committee on Cancer (AJCC) stage [17], hormone receptor status, tumor size, lymph node status, surgery type and radiation treatment; (iii) vital status; and (iv) diagnoses of multiple primary cancers in our cohort. Data on treatment with adjuvant chemotherapy and endocrine therapy were not available Further details and methods used by the SEER Program are provided elsewhere [18].

#### Second primary prostate cancer cases

Men were followed from their index first primary breast cancer diagnosis to the occurrence of a second primary cancer, death or end of the study period (December 2012). All second primary cancers were diagnosed among index breast cancer cases between 1988 and 2012. Second primary cancers were defined based on previously established SEER criteria [18] requiring a second primary malignancy subsequent to the index first primary male breast cancer to be located in a site with a different International Classification of Diseases for Oncology third edition (ICD-O-3) code and not documented as a metastasis in the clinical record. Men with other second primary cancers identified within 6 months following the index breast cancer were considered synchronous tumors and excluded.

#### Standardized incidence ratios and excess risk

We estimated standardized incidence ratios (SIR) and 95% confidence intervals (CI) comparing the observed occurrence of second primary prostate cancer after first primary breast cancer to cancer incidence rates expected in an age-standardized population of men in the SEER region ascertainment areas using Poisson regression [19]. We determined expected incidence rates and person-years at risk for second primary prostate cancer in stratum of 5-year intervals for age and calendar-year. These expected cancer incidence rates were then estimated using the product of at risk person-years in each stratum and their corresponding standardized incidence rates. These products were summed across strata of the five year intervals overall and in subgroup analyses. The reference standardized incidence rates for prostate cancer accounted for age (5-year interval), calendar year (5-year interval) and geographic location (SEER reporting region). We further conducted stratified sub-analyses to determine stratum-specific SIRs by age group, race/ethnicity, breast cancer AJCC stage and hormone receptor status. We calculated the overall and stratum-specific absolute excess risk of second primary prostate cancer as the difference between number of observed and expected cases divided by person-years at risk and report these estimates per 10,000 person-years.

#### Risk of second primary prostate cancer and mortality

Men were followed for these secondary endpoints from diagnosis to the date of death or end of the study period in December 2013. We separately examined cause-specific hazards of second primary prostate cancer and death due to breast cancer [20]. Men entered the analysis once they were considered at risk for secondary primary cancer at a left truncated entry time (6 months post-breast cancer) and contributed person-time until the first of death, second primary prostate cancer, other second primary cancer or end of the study period. Men who died from other causes or were lost to follow-up were considered censored at the time of loss or death in cause-specific analyses.

Using multivariable Cox proportional hazards models, we estimated hazard ratios (HR) with 95% CI for associations with the risk of second primary prostate cancer, breast cancer-specific mortality and all-cause mortality. We determined these associations in models adjusting for year of diagnosis and SEER registry; and in fully multivariable-adjusted models with adjustment for potential confounders selected a priori: age (20–55, 55–64, 65–74, 75+ years), AJCC stage (I, II, III) race/ethnicity (non-Hispanic White, Black, Asian/Pacific Islander, Hispanic, unknown) hormone receptor status (ER+/PR+, ER+/PR–, ER−/PR+, ER−/PR–, unknown), tumor size (<2 cm, ≥2 cm, unknown), lymph node status (negative, positive, unknown), surgery performed (yes/no) and radiation (yes/no).

The proportional hazards assumption was evaluated by plotting the logarithm of the negative logarithm of the survival function over time. We found no evidence contradicting the proportionality assumption. All analyses were performed using SEER*Stat [16] and SAS statistical software version 9.3 (SAS Institute Inc., Cary, North Carolina).

## Results

Descriptive characteristics of first primary male breast cancer cases are summarized in Table 1. Of the 5753 men diagnosed with first primary breast cancer included in the analysis, 75% were Non-Hispanic White, 13% were Black, 6% were Hispanic, and 5% were Asian/Pacific Islander. Median age at diagnosis was 66 years and most male breast cancers were stage I (30%) or stage II (37%), ER and/or PR positive (76%), tumor size <2 cm (68%) and lymph node negative (52%). Most men received surgery for their primary breast cancer (91%) while only a smaller proportion was treated with radiation (24%). After a median follow up of 4.3 years, 250 s primary prostate cancers were diagnosed. The median latency period between breast cancer diagnosis and second primary prostate cancer was 36 months (Table 2). Most second primary prostate cancers were stages I-II (50%). Men ages <70 years were more often diagnosed with stages I-II prostate cancer compared to men 70+ years (58% vs. 36%, *P* = 0.003).

**Table 1:** Descriptive characteristics of first primary male breast cancer cases from the SEER 18 registries, 1988–2012 (*N* = 5753)

	n	(%)
Age at index breast cancer, years		
Mean (SD)	65.2	(12.6)
Median (interquartile range)	66	(56–75)
20–44	334	(5.8)
45–54	869	(15.1)
55–64	1447	(25.2)
65–74	1607	(27.9)
75+	1496	(26.0)
Race/ethnicity		
Non-Hispanic White	4291	(74.6)
Black	757	(13.2)
Asian/Pacific Islander/Alaska Native	311	(5.4)
Hispanic	340	(5.9)
Unknown	54	
Breast cancer AJCC stage		
I	1696	(29.5)
II	2145	(37.3)
III	951	(16.5)
Missing/unknown	961	
Hormone receptor status		
ER/PR negative	213	(3.7)
ER and/or PR positive	4373	(76.0)
Unknown	1167	
Tumor size		
<2 cm	3923	(68.2)
≥2 cm	1348	(23.4)
Missing	482	
Lymph node status		
Negative	2982	(51.8)
Positive	2626	(23.4)
Missing	482	
Surgery		
Surgery performed	5210	(90.6)
Refused or not recommended	466	(8.1)
Missing/unknown	77	
Radiation		
No	4199	(73.0)
Yes	1371	(23.8)
Unknown	183	
Outcome status		
Second primary prostate cancer	250	(4.3)
Other second primary cancer	648	(11.3)
Vital status		
Alive	3312	(57.6)
Died (any cause)	2441	(42.4)
Cancer-specific death	1080	(18.8)
Person-years of follow up		
Mean (SD)	5.6	(4.9)
Median (interquartile range)	4.3	(1.8–8.4)

**Table 2:** Descriptive characteristics of second primary prostate cancer cases by age at index breast cancer from the SEER 18 registries, 1988–2012

	All cases (*N* = 250)		Age < 70 years at index breast cancer (*n* = 156)		Age 70+ years at index breast cancer (*n* = 94)		
	n	(%)	n	(%)	n	(%)	*P* value*
Age at second primary prostate cancer, years							
Mean (SD)	70.9	(8.1)					
Race/ethnicity							
Non-Hispanic White	192	(76.8)	119	(76.3)	73	(77.7)	0.893
Black	39	(15.6)	26	(16.7)	13	(13.8)	
Asian/Pacific Islander/Alaska Native	7	(2.8)	4	(2.6)	3	(3.2)	
Hispanic	12	(4.8)	7	(4.5)	5	(5.3)	
Latency period to prostate cancer diagnosis, months							
Median (interquartile range)	36	(14–84)	47	(17–103)	28	(10–57)	<0.001
Prostate cancer AJCC stage							
I	2	(0.8)	1	(0.6)	1	(1.1)	0.003
II	122	(48.8)	89	(57.1)	33	(35.1)	
III	12	(4.8)	10	(6.4)	2	(2.1)	
IV	18	(7.2)	6	(3.8)	12	(12.8)	
Unknown	96		50		46		

*To test for differences between groups we used Fisher’s exact test for categorical variables with cells <5 and Wilcoxon rank-sum test for medians

Overall, the incidence of second primary prostate cancer was non-significantly increased (SIR = 1.12, 95% CI 0.93–1.33) compared to the age-matched general population (Table 3). Incidence of second primary prostate cancer was increased in men ages 65–74 years at index breast cancer (SIR = 1.34, 95% CI 1.01–1.73) with an excess of 30 cases per 10,000 person-years; whereas incidence was lower in men ages 75+ years (SIR = 0.54, 95% CI 0.29–0.93, −34 cases per 10,000 person-years). Incidence of second primary prostate cancer was also higher in men diagnosed with AJCC stage I (SIR = 1.36, 95% CI 1.04–1.75) and hormone receptor-positive breast cancer (SIR = 1.23, 95% CI 1.11–1.39) with an excess of 24 and 22 cases per 10,000 person-years respectively.

**Table 3:** Standardized incidence ratios (SIR) and 95% confidence intervals (CI) for risk of second primary prostate cancer and absolute excess risk per 10,000 person-years among male breast cancer cases from the SEER 18 registries, 1988–2012

	SIR	(95% CI)	Excess risk per 10,000	Mean person-years at risk
Overall	1.12	(0.93–1.33)	7.65	6.05
Stratified estimates				
Age at index breast cancer, years				
20–55	1.22	(0.68–2.01)	5.94	7.41
55–64	1.20	(0.87–1.62)	14.77	6.64
65–74	1.34	(1.01–1.73)	30.28	6.17
75+	0.54	(0.29–0.93)	−34.17	4.28
Race/ethnicity				
Non-Hispanic White	1.12	(0.91–1.37)	8.33	6.23
Black	1.14	(0.68–1.81)	12.28	5.07
Asian/Pacific Islander/Alaska Native	1.16	(0.38–2.70)	5.88	6.35
Hispanic	1.01	(0.33–2.35)	0.32	5.31
Breast cancer AJCC stage				
I	1.36	(1.04–1.75)	23.70	7.25
II	1.17	(0.87–1.54)	11.18	6.04
III	0.71	(0.36–1.28)	−19.29	4.83
Hormone receptor status				
ER/PR negative	0.26	(0.01–1.44)^a^	−47.13	6.04
ER and/or PR positive	1.23	(1.11–1.39)	22.11	7.27

^a^Estimate based on <5 observed cases

In multivariable analyses among men diagnosed with primary breast cancer (Table 4), risk of second primary prostate cancer was increased with older age (55–64 years: HR = 3.69, 95% CI 2.04–6.67; 65–74 years: HR = 5.08, 95% CI 2.85–9.06; 75+ years: HR = 2.70, 95% CI 1.39–5.24) compared to younger men ages <55 years. The age of 55 was chosen as a cut off because this is the age that prostate cancer screening would be recommended for average risk men. Findings were suggestive of increased risk of second primary prostate cancer in Black men (HR = 1.38, 95% CI 0.86–2.24) compared to non-Hispanic White men but this was not statistically significant. Similarly, Black men were at increased risk of cancer-specific mortality (HR = 1.70, 95% CI 1.31–2.20) and all-cause mortality (HR = 1.56, 95% CI 1.31–1.87) following primary breast cancer compared to non-Hispanic White men (Table 5).

**Table 4:** Hazard ratios (HR) and 95% confidence intervals (CI) for breast cancer characteristics in relation to risk of second primary prostate cancer

	Crude model^a^		Multivariable-adjusted model^b^	
	HR	(95% CI)	HR	(95% CI)
Age at index breast cancer, years				
<55		Reference		Reference
55–64	3.41	(2.12–5.48)	3.69	(2.04–6.67)
65–74	4.34	(2.73–6.89)	5.08	(2.85–9.06)
75+	2.75	(1.63–4.62)	2.70	(1.39–5.24)
* P* trend		<0.001		0.001
Race/ethnicity				
Non-Hispanic White		Reference		Reference
Black	1.33	(0.92–1.91)	1.38	(0.86–2.24)
Asian/Pacific Islander/Alaska Native	0.55	(0.25–1.20)	0.47	(0.19–1.18)
Hispanic	0.93	(0.51–1.70)	0.77	(0.33–1.78)
Breast cancer AJCC stage				
I		Reference		Reference
II	0.85	(0.64–1.13)	1.10	(0.69–1.73)
III	0.64	(0.43–0.97)	0.84	(0.41–1.72)
Hormone receptor status				
ER/PR negative		Reference		Reference
ER and/or PR positive	1.29	(0.57–2.93)	2.41	(0.76–7.63)
Tumor size				
<2 cm		Reference		Reference
≥2 cm	0.75	(0.54–1.03)	0.69	(0.43–1.10)
Lymph node status				
Negative		Reference		Reference
Positive	0.73	(0.56–0.95)	0.73	(0.46–1.16)
Radiation				
No		Reference		Reference
Yes	0.96	(0.71–1.29)	1.14	(0.78–1.67)

^a^Crude model estimates adjusted for SEER registry and year of diagnosis only

^b^Multivariable-adjusted model estimates adjusted for age, race/ethnicity, breast cancer AJCC stage, hormone receptor status, tumor size, lymph node status, radiation, surgery, SEER registry and year of diagnosis

**Table 5:** Hazard ratios (HR) and 95% confidence intervals (CI) for breast cancer characteristics in relation to risk of cancer-specific mortality and overall mortality

	Breast cancer-specific mortality				Overall mortality			
	Crude model^a^		Multivariable-adjusted model^b^		Crude model^a^		Multivariable-adjusted model^b^	
	HR	(95% CI)	HR	(95% CI)	HR	(95% CI)	HR	(95% CI)
Age at index breast cancer, years								
<55	0.77	(0.64–0.92)	0.70	(0.53–0.91)	0.25	(0.22–0.28)	0.20	(0.17–0.24)
55–64	0.75	(0.63–0.90)	0.63	(0.48–0.82)	0.31	(0.28–0.35)	0.25	(0.22–0.30)
65–74	0.68	(0.57–0.81)	0.71	(0.55–0.92)	0.44	(0.39–0.48)	0.44	(0.39–0.51)
75+		Reference		Reference		Reference		Reference
* P* trend		<0.001		0.014		<0.001		<0.001
Race/ethnicity								
Non-Hispanic White		Reference		Reference		Reference		Reference
Black	1.82	(1.54–2.15)	1.70	(1.31–2.20)	1.34	(0.19–1.52)	1.56	(1.31–1.87)
Asian/Pacific Islander/Alaska Native	0.99	(0.71–1.37)	0.97	(0.60–1.59)	0.77	(0.62–0.97)	0.73	(0.53–0.99)
Hispanic	1.10	(0.83–1.48)	1.27	(0.85–1.91)	0.93	(0.76–1.13)	1.12	(0.86–1.47)
Breast cancer AJCC stage								
I		Reference		Reference		Reference		Reference
II	2.53	(2.01–3.17)	1.44	(1.01–2.03)	1.47	(1.31–1.65)	1.31	(1.09–1.56)
III	6.75	(5.36–8.49)	3.18	(2.12–4.77)	2.48	(2.18–2.82)	2.34	(1.86–2.96)
Hormone receptor status								
ER/PR negative		Reference		Reference		Reference		Reference
ER and/or PR positive	0.51	(0.39–0.67)	0.71	(0.48–1.05)	0.78	(0.63–0.96)	0.86	(0.65–1.13)
Tumor size								
<2 cm		Reference		Reference		Reference		Reference
≥2 cm	2.90	(2.40–3.50)	1.67	(1.27–2.19)	1.85	(1.66–2.06)	1.22	(1.04–1.44)
Lymph node status								
Negative		Reference		Reference		Reference		Reference
Positive	3.49	(3.04–4.02)	1.91	(1.46–2.51)	1.54	(1.42–1.68)	1.12	(0.96–1.31)
Radiation								
No		Reference		Reference		Reference		Reference
Yes	1.59	(1.38–1.81)	0.83	(0.68–1.02)	1.10	(1.00–1.21)	0.77	(0.68–0.89)

^a^Crude model estimates adjusted for SEER registry and year of diagnosis only

^b^Multivariable-adjusted model estimates adjusted for age, race/ethnicity, breast cancer AJCC stage, hormone receptor status, tumor size, lymph node status, radiation, surgery, SEER registry and year of diagnosis

## Discussion

Our results indicate a non-significant increase in incidence of second primary prostate cancer (SIR = 1.12, 95% CI 0.93–1.33) in men with breast cancer, which is in agreement with the prior 2002 SEER study [15]. On the other hand, the current study did not confirm the higher incidence demonstrated in the international cancer registry study (SIR = 1.61, 95% CI 1.34–1.93) [13]. These differences may be attributable to prostate cancer screening patterns, variability given the small numbers of cases, or geographical differences. Given the rarity of male breast cancer, large cohorts such as population-based databases are often utilized. Our study represents the largest analysis of this population to date with 250 cases of second primary prostate cancer. Although the number of second primary prostate cancer cases in this analysis is still modest, there were a sufficient number of cases to allow subset analyses of patient and tumor characteristics that have not been preformed previously.

An increased incidence of prostate cancer in the male breast cancer population was significant in men ages 65–74 (SIR = 1.34, 95% CI 1.01–1.73) with an excess of 30 cases per 10,000 person-years. Conversely, in men >75 years the incidence ratio and hazard ratios are lower. This apparent protective-effect with older age may be an artifact due to a detection bias as a result of decreased PSA screening in this population due to advanced age, in addition to a known cancer diagnosis. This detection bias may also in part explain the low rate of prostate cancer in this cohort, since men over age 75 account for 26% of breast cancer cases. Men with stage AJCC stage 1 of breast cancer also had an increased incidence for developing prostate cancer (SIR = 1.36, 95% CI 1.04–1.75). This was not seen in the higher stages of breast cancer. This may similarly represent a detection bias or a survival bias due to higher mortality rates in higher stages [21]. In these men higher stage breast cancer represents a competing risk for mortality. Hormone receptor positivity was also associated with a significantly higher SIR for developing prostate cancer. This may be a detection bias and survival bias, as a result of the poorer survival in ER negative patients [21]. Our data suggests that prostate cancer screening may be indicated in men with stage 1 breast cancer, hormone receptor-positive breast tumors, and men diagnosed with breast cancer who are over 65.

The apparent association between male breast and prostate cancer could be due to environmental, biologic or genetic similarities between the two diseases. Estrogen plays an important role in both of these cancers [22, 23]. In addition, androgens have been found to have a role in male breast cancer [24, 25] and are well known to have important effects on prostate cancer [26]. It has also been postulated that glucocorticoid receptors have an important role in both cancers [27]. Therapies for each include hormone manipulation, which may in turn have some bearing on the risk for subsequent cancers. Therefore, the existence of a common risk factor(s) for these two diseases is biologically plausible.

The use of a population-based database has several limitations. Individual-level information was limited. While data were available on many tumor characteristics from SEER, we did not have information on other tumor markers, family history, sexual orientation, karyotype, hormonal levels, lifestyle characteristics and genetic factors including a lack of information on results of DNA testing for germline mutations in the *BRCA* genes. There is a known association with *BRCA* and both breast and prostate cancer [6, 7], lack of this data could be a confounding factor. However we believe this to be modest as *BRCA* positive patients represent a small proportion of all male breast cancer patients [8]. Gleason score was also unavailable in SEER for the majority of the prostate cancer cohort and therefore could not be included in the analysis. It would be useful to determine whether second primary prostate cancers have a distinctive and/or aggressive biology.

Despite these limitations, our study has several strengths including longer-term follow up after male breast cancer compared with previous studies, information on multiple other primary cancers and death as competing events for second primary prostate cancer, and a greater number of male breast cancer cases from multiple geographically-diverse, population-based cancer registries in the U.S.

## Conclusion

Overall for men with breast cancer, no increased risk of developing prostate cancer has been demonstrated. However, this analysis suggests a possible association between early-stage, hormone-sensitive male breast cancers and subsequent primary prostate cancer. This finding may be confounded by the risk of detection bias in this group of patients with these positive prognostic indicators.

## Abbreviations

AJCC: American Joint Committee on Cancer
BRCA: Breast cancer susceptibility gene
CI: Confidence interval
DNA: Deoxyribonucleic acid
ER: Estrogen receptor
HR: Hazard ratio
PR: Progesterone receptor
PSA: Prostate specific antigen
SEER: Surveillance, Epidemiology and End Results Program
SIR: Standardized incidence ratio
